# An ice inhabiting bdelloid rotifer from North America

**DOI:** 10.1007/s00792-025-01390-6

**Published:** 2025-07-10

**Authors:** Lydia M. Dimattia, Naim Saglam, Ralph Saunders, Daniel H. Shain

**Affiliations:** 1 Biology Department, Rutgers The State University of New Jersey, Camden, NJ 08103 USA; 2 Department of Aquaculture and Fish Diseases, Fisheries Faculty, Firat University, 23119 Elazig, Türkiye

**Keywords:** Glacier, Pleistocene, Pacific Northwest, Psychrophile, Cold adaptation, Metagenomics, Intraguild predation

## Abstract

Bdelloid rotifers are major components of zooplankton worldwide and have been reported in glacier ice in both Northern and Southern Hemispheres. Curiously, no reports of psychrophilic bdelloids have surfaced in North America despite exhaustive surveys of other ice-dwelling invertebrates, e.g., glacier ice worms. This distribution gap may be partially explained by a predator-prey relationship between these two animals, but the current study suggests that ice worms and bdelloids can co-inhabit at least some glacial ecosystems over geological time. Here we report the first ice-inhabiting bdelloid rotifer from North America, collected from the northern aspect of Mt. Deception, WA, USA. Nuclear and mitochondrial genotyping identified sister-species relationships within a clade of Nordic ice-dwelling bdelloids, and close evolutionary relationships with Antarctic/New Zealand specimens. Intrapopulation genetic divergences suggest that bdelloids arrived in North America near the onset of the Pleistocene (2.58 myr BP), but their circumpolar dispersal capabilities and robustness (e.g., freeze-thaw tolerance, ability to propagate at elevated temperatures and under extreme laboratory conditions) cannot rule out multiple transoceanic dispersal events throughout the Quaternary.

**Supplementary Information:**

The online version contains supplementary material available at 10.1007/s00792-025-01390-6.

## Introduction

Ice-dwelling microinvertebrates have been identified from Arctic/sub-Arctic regions as well as parts of Antarctica and New Zealand (Iakovenko et al. 2015; Shain et al. 2021, 2024). The dominant taxa in these icy habitats are primarily Rotifera and Tardigrada (Zawierucha et al. 2020), but species of Arthropoda (e.g., copepods, mites, collembola), Nematoda and Platyhelminthes are also reported (Shain et al. 2021). The only macroscopic metazoans know to reside permanently “in” glacier ice (i.e., spaces between ice crystal boundaries, as opposed to on the surface) are the ice worms, *Mesenchytraeus solifugus*, from maritime glaciers of North America (Tynen 1970), and *Sinenchytraeus glacialis*, from the Tibetan Plateau (Liang et al. 1979). Phylogenetic data suggest that many lineages of ice inhabiting Metazoa have persisted throughout the Pleistocene, and some appear to have a much deeper ancestry (Dial et al. 2012; Shain et al. 2021, 2024).

Bdelloidea Rotifera are known for their unusual biological properties. For example, this group has persisted for ~ 40 million years, reproducing primarily by parthenogenesis (Poinar and Ricci 1992; Fontaneto and Barraclough 2015; Laine et al. 2022). Many species are known to acquire genetic diversity by extensive horizontal gene transfer (Gladyshev et al. 2008; Boschetti et al. 2012; Flot et al. 2013), others undergo anhydrobiosis to survive desiccation or to escape fungal parasites (Ricci and Caprioli 2005; Wilson and Sherman 2010), and some resist extreme levels of ionizing radiation (Gladyshev and Meselson 2008). Collectively, more than 400 species of bdelloid rotifers are described and are known worldwide as major components of zooplankton in freshwater and terrestrial (moist soil, moss) habitats, with a few described marine species (Fontaneto et al. 2006; Segers 2007; Vñrstan and Plewka 2017).

Ice inhabiting bdelloids found above the equilibrium line altitude (ELA; without cryoconite holes) were first reported from Icelandic glaciers (Shain et al. 2016), and thereafter from New Zealand’s Southern Alps and throughout Norway, including Svalbard (Shain et al. 2021, 2024). A global phylogeny supports a monophyletic clade of ice bdelloids comprising ~ 20 species with an ancestry in the High Arctic dating to the mid-Miocene (~ 15 mya; Shain et al. 2024). Curiously, bdelloids have not been found in collections from ice worm-inhabiting glaciers in North America including Mt. Rainier, WA, The Three Sisters, OR and Chugach Range, AK. Despite these distribution gaps within suitable habitats of the Pacific Northwest (PNW), we report the first collection of ice-bdelloids from North America occurring in glacier ice from Mt. Deception, WA. Phylogenetic data aligns them most closely with Nordic ice-dwelling bdelloid species, and intrapopulation divergences suggest they have been in North America for most of the Quaternary.

## Materials and methods

### Specimen collection

Surface ice/snow from the northern aspect of Mt. Deception, WA (47.81693, -123.22828) containing glacier ice worms was collected on September 26, 2016. The sample was express-mailed to Rutgers University in an unused Tupperware container cooled with ice, transferred into a 500 ml beaker with aluminum foil cover and stored in a 4˚C walk-in cooler without lighting. In May 2021, a small aliquot of the remaining ~ 50 ml meltwater was viewed under a stereomicroscope revealing the presence of bdelloid rotifers (~ 10 individuals/ml). Over Summer 2021, ice worm collections were made at Byron Glacier, AK, Nisqually Glacier (Mt. Rainier, WA) and the South Sister, OR. All glacial meltwater from collections were screened exhaustively for microinvertebrates.

### Specimen processing

Sample meltwater from glacial collections is routinely stored in sterilized laboratory beakers in which ice worms survive 2 + years at 4˚C in darkness (Hartzell et al. 2005). Once ice worms have terminated, meltwater is usually discarded. Inadvertently, a set of beakers/collection bags, including glacial meltwater from a September 16, 2016, Mt. Deception, WA ice worm collection, were overlooked (along with other PNW collections) and remained in a walk-in cooler corner shelf at ~ 4˚C for ~ 5 years in darkness, without the addition of an external carbon source. In May 2021, ~ 50 ml of meltwater remained, comprising a slurry of sediment and suspended debris (Suppl. Figure 1). Prior to discarding contents of the beaker, it was viewed directly by stereomicroscopy; unexpectedly, a robust population of bdelloid rotifers was observed, mostly non-swimming, occurring on the bottom surface at densities up to ~ 100/cm^2^. Rotifers were not found in comparable beakers/collection bags from other collections in the PNW stored for similar time periods. To observe specimens, ~ 10 ml of glacial water was retrieved with a sterile pipet and viewed by stereomicroscopy. Individual rotifers were captured in 1–3 µl of field water using a fine pipet, transferred into 7 µl of 95% EtOH and stored at − 20 °C.

### DNA sequencing

Gene loci encoding cytochrome *c* oxidase subunit 1 (COI) and 18 S rRNA were targeted to gain insight into the evolutionary history of Mt. Deception rotifers. To extract DNA, EtOH was evaporated on a 65 °C heat block for ~ 5 min with lid open, then 10 µl of a solution containing 25 mM Tris pH 8.5, 50 mM KCl, 5 mM MgCl_2_ and proteinase K (20 µg/µl) was added. Following incubation at 55 °C for 20 min, proteinase K was inactivated at 95 °C for 2 min, spun briefly and 1 µl was removed for polymerase chain reaction (PCR) analysis. PCR reactions contained 1X DreamTaq mix (ThermoFisher), 0.4 mM respective primers [(COI; Folmer et al. 1994) and 18 S rRNA (Horton et al. 2017)] and 1 µl template in total reaction volume of 25 µl. Conditions for PCR were 95 °C for 2 min, then 94 °C (20 s) / 50 °C for COI, 54 °C for 18 S (40 s) / 72 °C (45 s) for 35 cycles, then 72 °C for 5 min. For DNA sequencing, PCR reactions were cleaned with a GeneJet Purification Kit (ThermoScientific) and sent to Azenta Life Sciences (South Plainfield, NJ) for Sanger sequencing using PCR primers on both strands accordingly.

### Phylogenetic analysis

Individual gene sequences for COI and 18 S rRNA were aligned using the MUSCLE algorithm (Edgar 2004) with default settings, as integrated into MEGA X v10 software (Kumar et al. 2018). Statistical analyses were conducted in MEGA to assess the phylogenetic informativeness of the dataset. Phylogenetic reconstructions were performed using Maximum Likelihood (ML) with 735 bp from 18 S rRNA and 461 bp from COI. Positions containing gaps or missing data were excluded from the final dataset. Datasets were analysed using the model selection module in MEGA X v10 to identify the optimal substitution model for ML-based phylogenetic tree construction. Based on the Bayesian Information Criterion (BIC) and corrected Akaike Information Criterion (AICc) scores, the best-fitting model was selected and used to generate the ML phylogenetic tree for the gene region. The General Time Reversible model with a Gamma distribution and invariant sites (GTR + G + I) was identified as the most suitable model for reconstructing phylogenies (Nei and Kumar 2000). The phylogenetic tree with the highest log-likelihood value was visualized, and the percentage of trees in which associated taxa clustered together was indicated on branches. Initial trees for the heuristic search were automatically generated using Neighbor-Joining and BioNJ algorithms. A discrete Gamma distribution was applied to account for rate variation across sites (Kumar et al. 2018). Haplotype maps were created by HaplowebMaker using default parameters (delimiter, mask error, radius proportional) with TCS software (Clement et al. 2000; Spöri and Flot 2020).

### Thermal tolerance analyses

Freeze-thaw experiments were conducted at -20˚C (freeze) and 4˚C (thaw) as described (Shain et al. 2024), to determine thermal tolerances of Mt. Deception rotifers. Growth was quantitated by monitoring three independent petri dishes with at least three rotifer specimens per dish at each respective temperature (4˚, 22˚, 27˚, 37˚C). Rotifer numbers in each dish were recorded daily.

### Metagenome analysis

Metagenomic whole genome shotgun (mWGS) sequencing was employed to characterize microbial communities in glacial meltwater, using 1 ml aliquots of sediment and aqueous phases, respectively. Samples were prepared with a ZymoBiomics DNA Microprep kit, Novogene (Sacramento, CA) performed the amplicon metagenomics sequencing (WBI). Data processing and visualization were conducted with Krona software (Ondov et al. 2011).

## Results

**Fig. 1:**
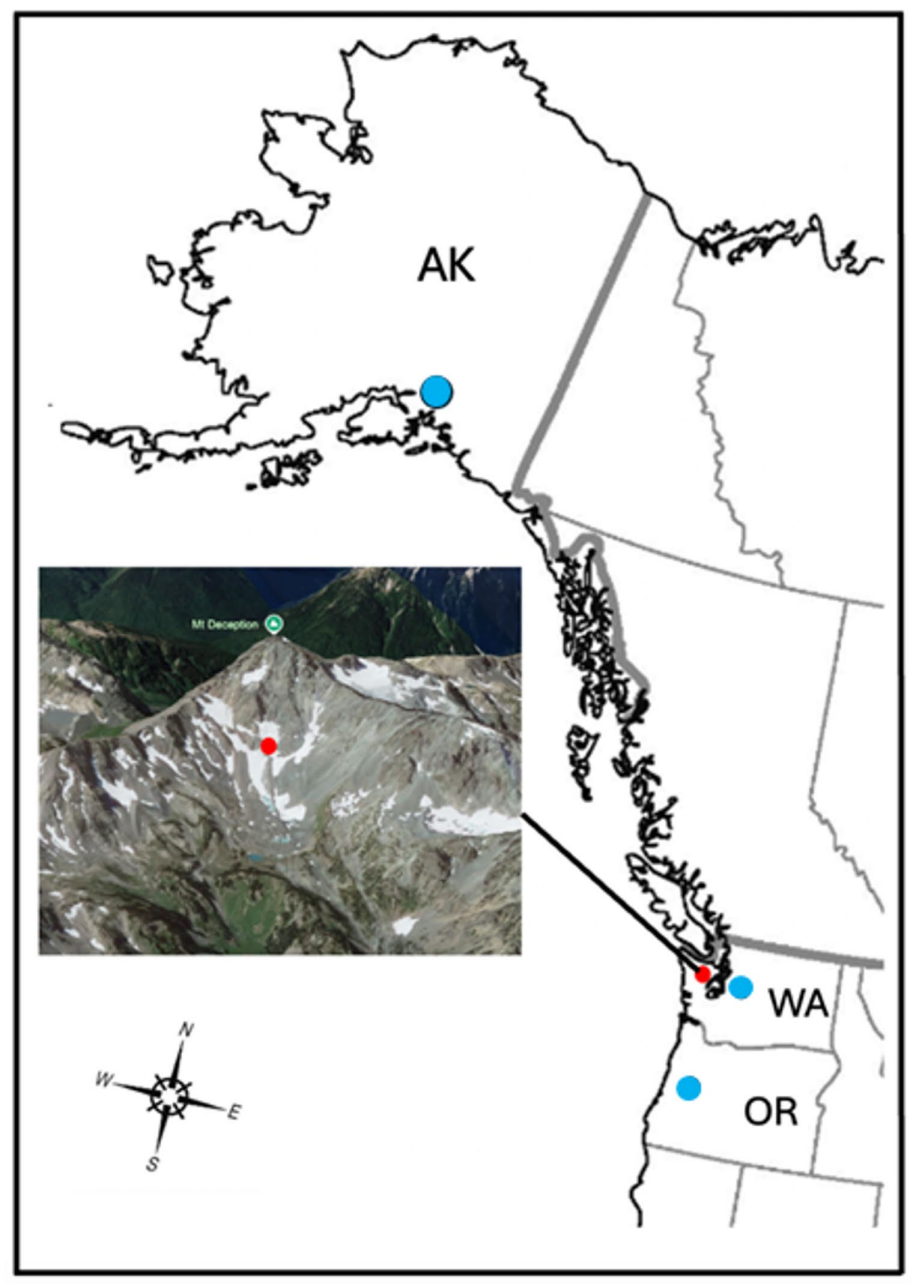
Glacier field sites visited. Red circle on Mt. Deception, OR identifies location (47.81693, -123.22828) at which glacial samples were collected and ice rotifers were found. Blue circles identify ice collections in which rotifers were not found (collections in Summer, 2021): Byron Glacier, AK; Nisqually Glacier, WA; South Sister Glacier, OR. All collections focused on ice worms feeding on the glacial surface, at least ~ 100 worms were collected at each field site in ~ 1 L equivalent of surface ice/snow

To date, glacier ice worm collections throughout the Pacific Northwest (e.g., Byron Glacier, AK, Three Sisters, OR, Nisqually Glacier, WA) have consistently failed to reveal co-occurring microinvertebrates (e.g., rotifers, tardigrades; Fig. 1). Unexpectedly, a robust rotifer community was observed in a shelved beaker stored at 4˚C for ~ 5 years containing glacial meltwater originating from Mt. Deception, WA. We can rule out laboratory contamination of this sample from foreign sources (e.g., Icelandic, New Zealand, Norwegian) because no other live rotifers were in culture prior to or during the processing of Mt. Deception specimens. Likewise, the possibility of intermixing between stored North American samples (e.g., airborne specimens in an anhydrobiotic state) seems unlikely since storage containers remained covered or sealed, and contents never dried out.

**Fig. 2 Note that this is a line diagram, increasing resolution is not applicable:**
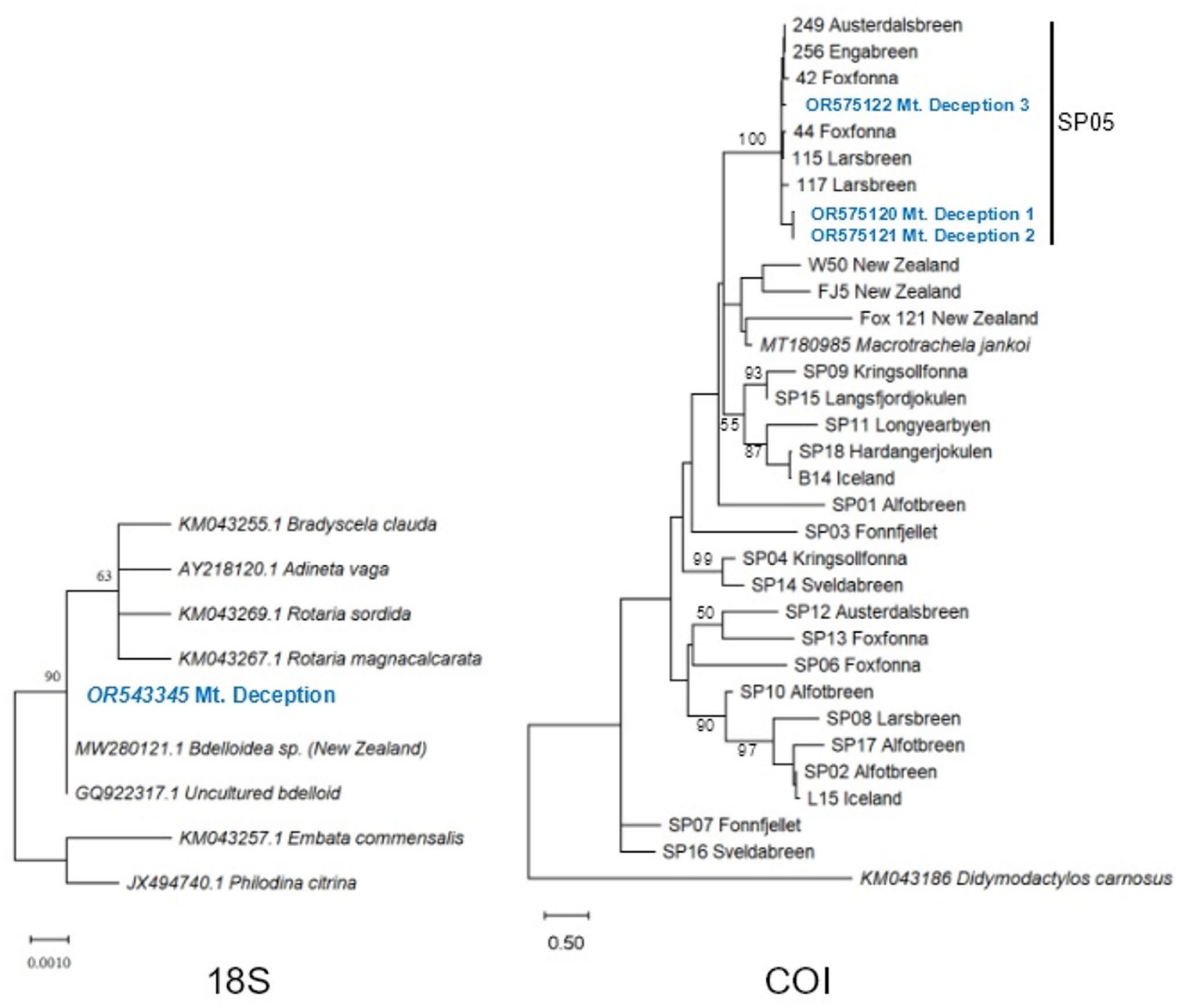
Bdelloid phylogeny at nuclear (18 S rRNA) and mitochondrial (COI) loci. Mt. Deception specimens (shown in blue) aligned with available 18 S bdelloid sequences from New Zealand in an unrooted tree (see Suppl. Figure 2 for rooted tree) and were nested within a Nordic clade designated SP05 (Shain et al. 2024) in an ice-bdelloid, global COI phylogeny (rooted with outgroup *Didymodactylos carnosus*). Based on mitochondrial divergence at COI (Tang et al. 2014), Mt. Deception haplotypes GenBank OR575120/1 and GenBank OR575122 are separated by 2–4 myr, and 6–10 myr from Antarctic/NZ bdelloids. Posterior probabilities shown at nodes accordingly

**Fig. 3:**
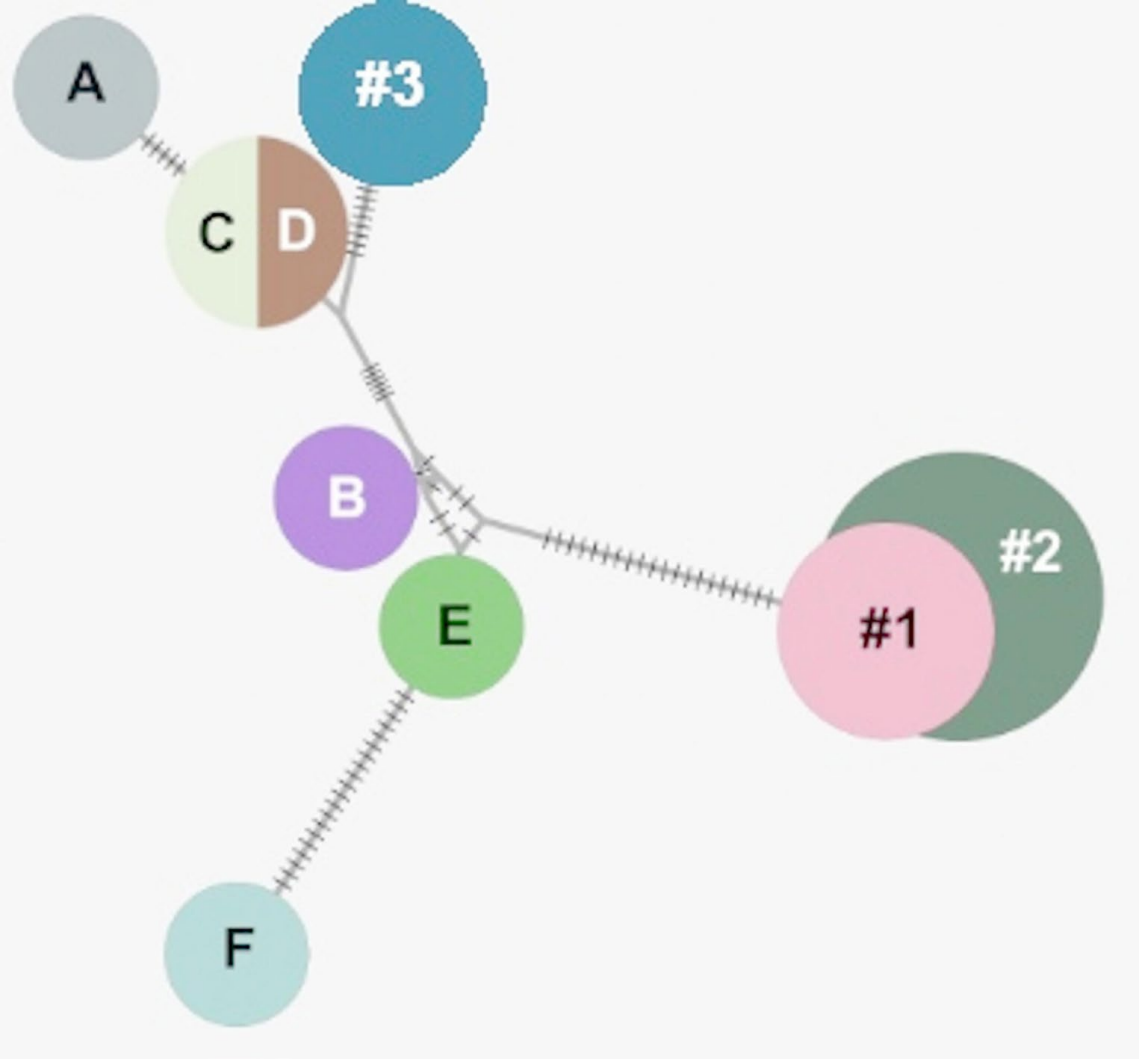
COI haplotype map of ice-inhabiting North American bdelloids and Norwegian congeners. Three variants (1, 2 and 3) were identified at Mt. Deception, WA, USA, aligning with specimens collected from different Norwegian glaciers, respectively (A-F). A, B – Foxfonna; C – Austerdalsbreen; D – Engabreen; E, F – Larsbreen

To assess the properties of Mt. Deception rotifers, a ~ 5 ml aliquot from the original beaker contents was transferred into a petri dish, from which random individuals were isolated. DNA was successfully extracted from 10 individuals, respectively, and subjected to PCR amplification at nuclear (18 S rRNA) and mitochondrial (COI) loci. Genetic distances from deposited sequences in GenBank suggested that specimens were undescribed species of Bdelloidea Rotifera (Fig. 2; Suppl. Figures 2–4). Intrapopulation divergence was monitored at the COI locus, identifying three variants with divergence values up to ~ 6% (Fig. 3; Suppl. Table 1). Their representation in the sample pool was five (designated Mt. Deception 1), three (Mt. Deception 2) and two (Mt. Deception 3). Applying a conservative molecular clock for rotiferan evolution of 1.76% per myr (Tang et al. 2014), an estimated maximum of ~ 2–4 myr separates the specimens included in our analysis. The broader COI phylogeny incorporating ice-inhabiting bdelloids reported worldwide aligned Mt. Deception bdelloids with a clade of Nordic specimens (SP05; Shain et al. 2024) collected from both mainland Norway and Svalbard (see Fig. 2).

**Fig. 4:**
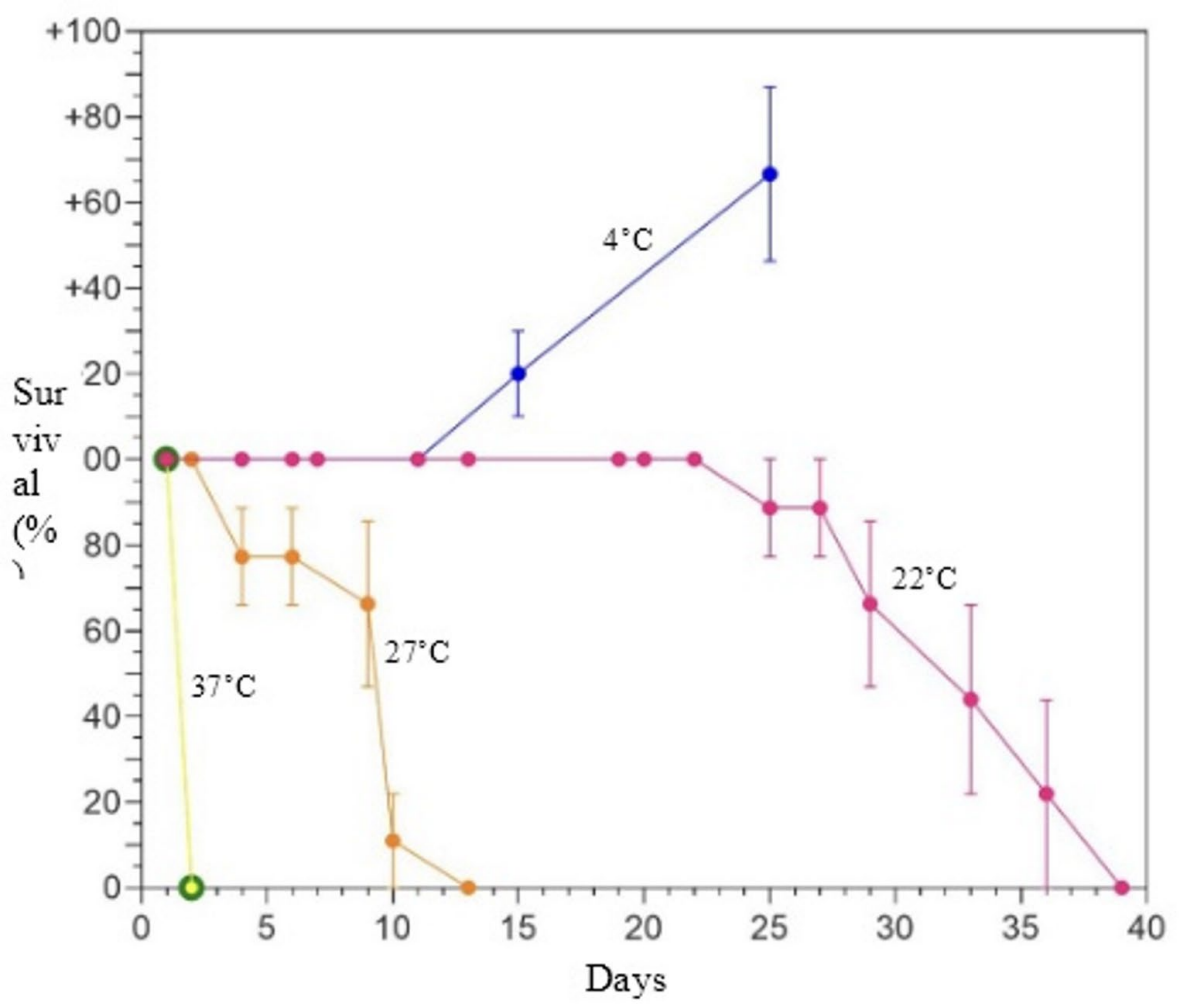
Thermal growth tolerance of bdelloid rotifers collected from Mt. Deception, WA. Specimens propagated at 4˚C but were not viable over longer time periods at 22˚C and above

To determine thermal tolerances of the Mt. Deception rotifers, specimens were frozen at -20˚C, and also cultured between 4–37˚C. Consistent with previous observations (Shain et al. 2024), ~ 75% of Mt. Deception rotifers survived a single freeze-thaw cycle at temperatures well below 0˚C (i.e., -20˚C). At temperatures above 0˚C, rotifers could propagate at 4˚C but declined gradually at 22˚C and sharply at 37˚C (Fig. 4).

**Fig. 5 Note that this is a line diagram, higher resolution is not applicable:**
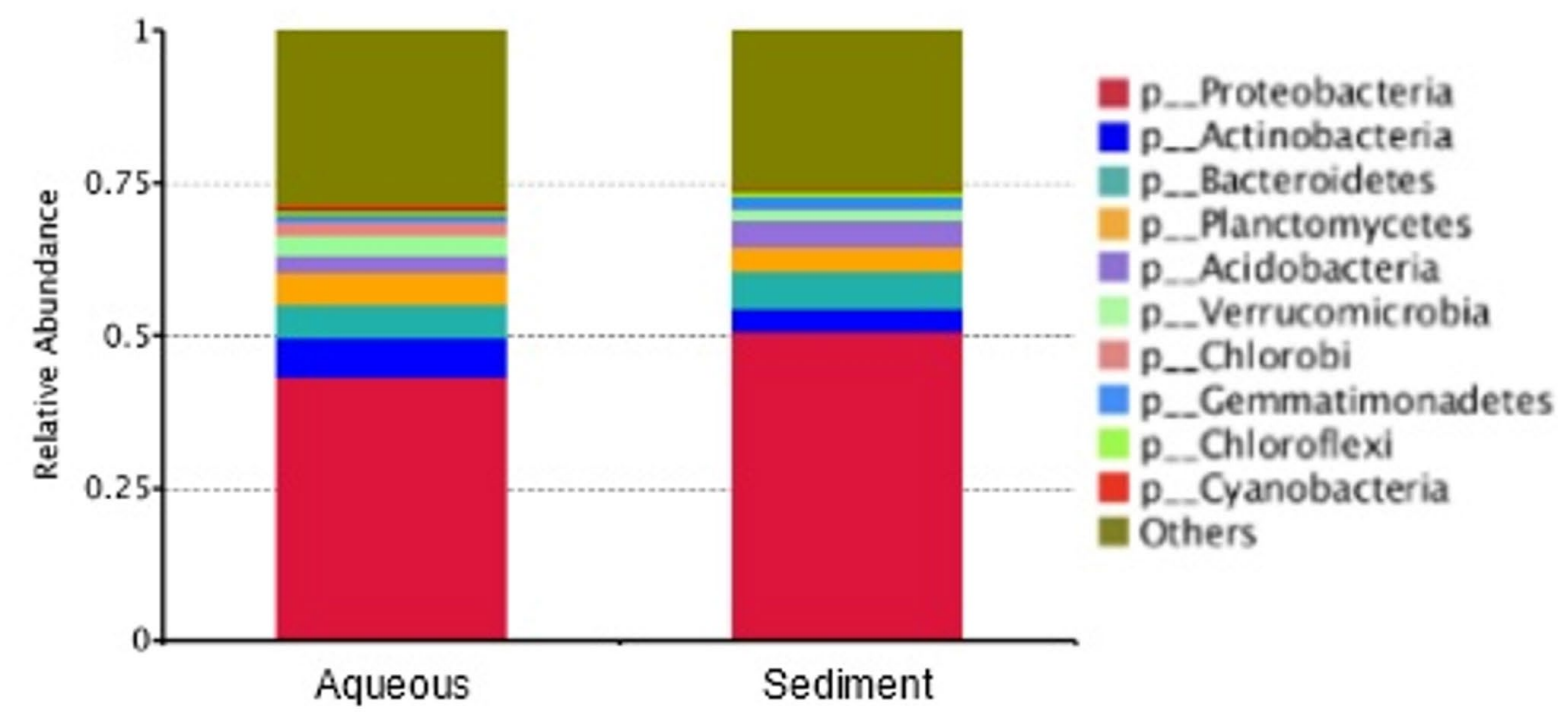
Prokaryotic composition of Mt. Deception beaker contents. Metagenomic profiling identified major bacterial phyla in aqueous and sediment aliquots. Proteobacteria were the most abundant bacteria in the microbial community

To determine the organismal composition of the original beaker contents–which was incubated for ~ 5 years in almost complete darkness at 4˚C without a supplemental carbon source–we generated metagenomes from 1 ml aliquots of sediment and aqueous phases, respectively. Profiles were comparable between the two samples, displaying a mixture comprising all three domains of life (Suppl. Figure 5). The bulk of identifiable biota was prokaryotic (~ 78%), of which the dominant taxa were Proteobacteria (~ 45%) with only a trace of Cyanobacteria (< 1%) (Fig. 5).

## Discussion

Bdelloid rotifers are known to occur at thermal extremes worldwide (Zawierucha et al. 2020; Shain et al. 2022), but reports from North American field sites (e.g., glaciers, thermal hot springs) are noticeably absent. The identification of ice-inhabiting bdelloids at Mt. Deception is informative for several reasons. First, maritime glacial ice to the North (e.g., Chugach Range, AK), South (Three Sisters) and East (Mt. Rainier, WA) do not appear to support rotifer populations; and secondly, our data suggests that bdelloids collected at Mt. Deception may have arrived in North America prior to or near the onset of glacial oscillations that define the Pleistocene (2.58 myr BP).

The apparent distribution gap of ice-inhabiting bdelloids in the PNW is not consistent with their widespread global distribution (Zawierucha et al. 2020; Shain et al. 2024), but may be explained by intraguild predation (Polis et al. 1989). In the context of food web dynamics, this region differs by the presence of a major natural competitor–and possible predator–in ice ecosystems, namely the glacier ice worm. Ice worms occur in most, and perhaps all, maritime glaciers along the PNW coastline between Oregon and southern Alaska (possible exceptions include Mt. Hood as a consequence of geologically recent volcanic activity), and at densities up to several hundred per square meter (Hartzell et al. 2005; Dial et al. 2012). As apex consumers in icy environments, ice worms indiscriminately ingest microbial biota on the glacial surface (e.g., algae, bacteria, fungi), a diet that is predictably similar to ice-inhabiting bdelloids. Since both ice worms and bdelloids graze on the same organic surface layer, it seems inevitable that ice worms–the only macroscopic organism known to reside in ice—would regularly ingest microbial bdelloids as part of their natural diet. If so, this falls into an intraguild predation model, a common dynamic within food webs that involves competing species with a common food source who are also locked in a predator-prey relationship, typically leading to declines in the prey population(s) (Polis and Holt 1992; Dial and Roughgarden 1995; Fedriani et al. 2000). While an asymmetric intraguild predation dynamic among ice worms (apex predator), rotifers (prey) and their common food source (glacial microbes) may explain the broad distribution gap of bdelloids in the PNW, it is not consistent with the co-occurrence of ice worms and bdelloids on Mt. Deception. Possibly the geomorphology of the latter contributes to this disparity; for example, fragmented ice on the northern aspect of Mt. Deception (see Fig. 1) may have microclimatic pockets that freeze solid overwinter (e.g., elevation differences, small ice patches or shallow edges), creating a maritime/continental ice continuum. Since ice worms are particularly sensitive to freezing temperatures (Edwards 1986), and bdelloids can survive through multiple freeze-thaw cycles (Shmakova 2021; Shain et al. 2016, 2024), different parts of the same glacial basin may permit the survival of both species concurrently over geological time.

Factoring the long-term instability of glacier ice in the Olympic Peninsula, the estimated time divergence (2–4 myr) between rotifer individuals at Mt. Deception suggests the occurrence of multiple, independent dispersal events and/or an ability to survive through climatic warm periods. Specifically, numerous interglacials throughout the Quaternary, most recently the Middle Holocene thermal maximum (7.5–4.2 ka BP), likely melted glacier ice throughout the Olympic Peninsula (Schafer et al. 2010; Clague and Ward 2011; Tzedakis et al. 2022). Thus, unless ice-dwelling bdelloids were able to survive and propagate in local ponds/streams at elevated temperatures, they would be subjected to local extinction(s) periodically over the past several million years. Our current data provides some plausibility for this alternating aquatic/ice life history (e.g., bdelloids can reproduce at 4˚C in laboratory cultures; see Fig. 4), but others have questioned this possibility based on the global absence of psychrophilic bdelloids in non-glacial terrain (Shain et al. 2024). Clearly, it will be instructive to sample non-glacial, aquatic habitats at Mt. Deception to determine the current distribution of the bdelloids in question. A competing hypothesis is that since ice-rotifers appear to disperse efficiently in circumpolar patterns, Arctic species may regularly populate glaciated regions throughout the Northern Hemisphere (e.g., Shain et al. 2016, 2024). In that scenario, Mt. Deception and other glacial habitats in the PNW may have been colonized recently (e.g., even following the Holocene), but ice worm predation has kept their numbers low and restricted to relatively few location(s) (e.g., Mt. Deception). Supporting this notion, some Mt. Deception specimens were more similar to Norwegian bdelloids than to each other (see Fig. 3). Thus, intrapopulation divergences observed in contemporary North American bdelloids could be derived from multiple, independent dispersal events.

Equally perplexing was the discovery of a robust rotifer population in a beaker stored for ~ 5 years in almost complete darkness, without a supplemental carbon source. We note that ice worms are capable of surviving in equivalent conditions for 2 + years in laboratory cultures (Hartzell et al. 2005), but they inevitably terminate without reproducing. Remarkably, the original Mt. Deception beaker is ongoing with live rotifers (currently ~ 8.5 years), supplemented occasionally with sterile water only to maintain volume. The microbial composition of this beaker “microenvironment” is surprisingly similar to other reported glacial metagenomes, with Proteobacteria comprising the bulk of representative species (Liu et al. 2022). Interestingly, Cyanobacteria—which could in principle fix atmospheric C and N in this closed community (Whitton and Potts 2012)—were observed at comparatively lower levels in the Mt. Deception beaker (~ 2.4% vs. < 1%) consistent with near dark conditions, and it seems unlikely that they are sustaining the robust bdelloid population. Possibly other non-photosynthetic, carbon-fixing microbes (e.g., Hu et al. 2015; Anand et al. 2020) have acquired a primary production service that sustains the microbial community, utilizing atmospheric CO_2_ as a carbon source. Our metagenomic profile does not provide resolution to provide further insights into this curious microenvironment.

Collectively, our data supports the successful colonization of Mt. Deception ice by bdelloid rotifers, likely dispersing from Nordic population(s) just prior to or during the Quaternary. A more precise timeframe of these events is difficult to determine due to the robustness of these organisms, namely they are freeze-tolerant, can propagate at elevated temperatures (i.e., > 0˚C) and appear capable of surviving in nutrient-limited, extreme environments under laboratory conditions. Thus, Mt. Deception bdelloids may have colonized regional PNW ice as recently as a few thousand years ago (i.e., following the Holocene thermal maximum), or could have persisted through multiple interglacials of the Pleistocene by surviving in ice refugia (e.g., ice caves, rock glaciers) or by alternating between ice and aquatic lifestyles.

## Electronic Supplementary Material

Below is the link to the electronic supplementary material.

Supplementary Material 1
