# Supplementary material for: An ice inhabiting bdelloid rotifer from North America
Source: Extremophiles. 2025 Jul 10;29(2):30. doi: 10.1007/s00792-025-01390-6 (PMC12246028; doi:10.1007/s00792-025-01390-6)
Supplement: Supplementary file 1 — Supplementary Material 1 [file 792_2025_1390_MOESM1_ESM.docx]

**Supplementary Figures**

**
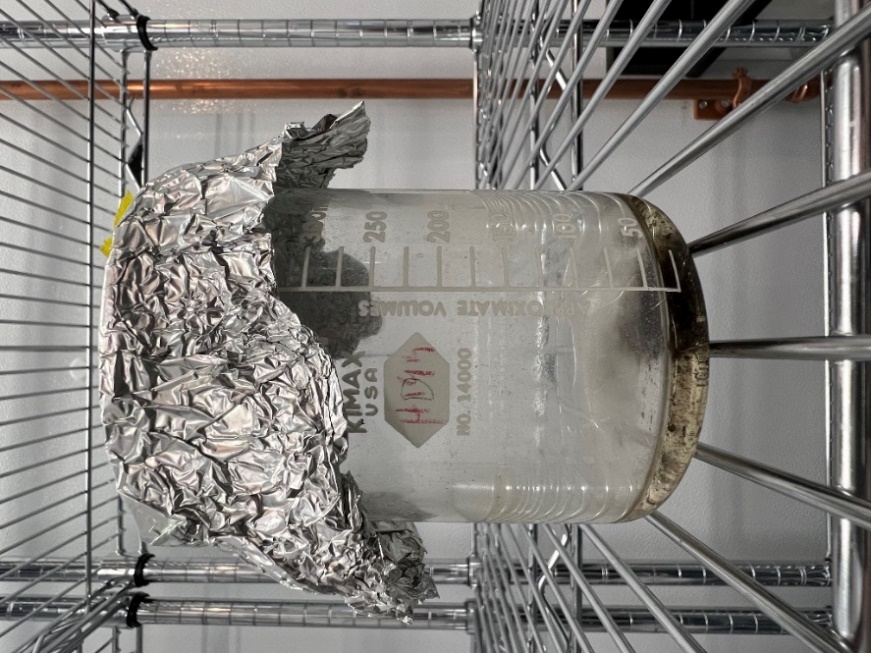
**

**Figure 1**. Original storage beaker with glacial meltwater from 9/16/2016 Mt. Deception ice worm collection. Beaker was inadvertently incubated for ~5 years at 4˚C in near darkness before rotifers in the current study were discovered. Contents of this beaker and other samples in the same proximity never dried out, thus minimizing the possibility of cross-contamination by airborne specimens (e.g., rotifers in a dehydrated, anhydrobiotic state). Likewise, no live rotifer specimens from Iceland, New Zealand or Norway were in culture prior to or during the time period in which Mt. Deception rotifers were analyzed.


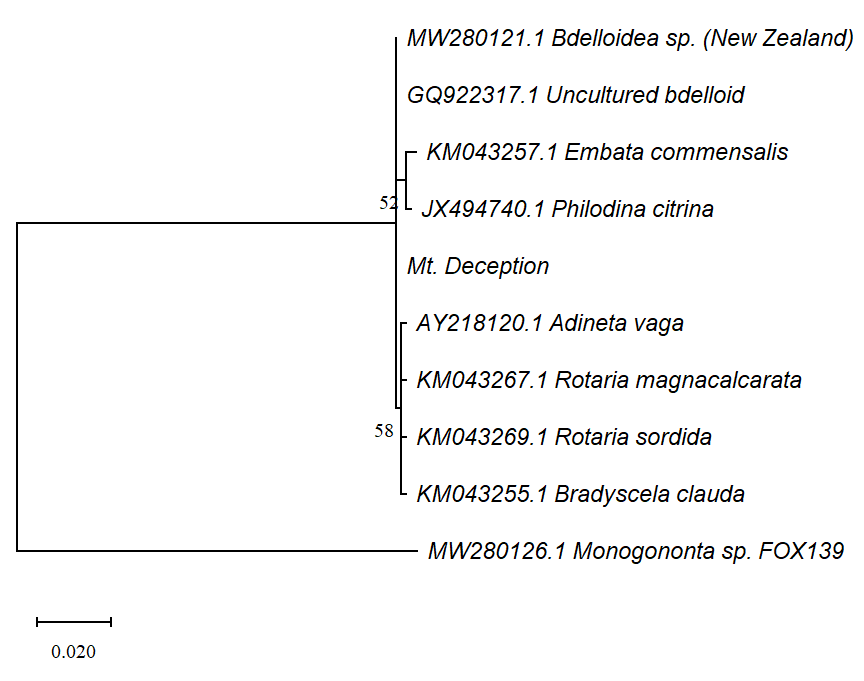


***OR543345* Mt. Deception**

**Figure 2.** Bdelloid phylogeny at the 18S rRNA locus. Mt. Deception variants 1-3 were identical within the 735 bp processed. Tree was rooted with a monogonont specimen collected from Fox Glacier, New Zealand. Posterior probabilities shown at nodes accordingly.

***Species Delimitation*** (Figs. 3 and 4, below)

The COI mtDNA gene region dataset of rotifers was utilized for species delimitation analysis. Two different methods were employed: ASAP (Assemble Species by Automatic Partitioning; Puillandre et al. 2021) and GMYC (General Mixed Yule-Coalescent; Fujisawa & Barraclough 2013). To perform the analyses, sequences in the COI dataset were aligned using BioEdit v7.2.5 software (Hall 1999). For ASAP, aligned sequences were uploaded to the https://bioinfo.mnhn.fr/abi/public/asap/ website, where appropriate parameters were set to complete the process. For GMYC, the aligned dataset was evaluated using MEGA X software to identify the best-fitting model based on Maximum Likelihood. The General Time Reversible (GTR) Gamma substitution was selected as the best-fitting model, and an XML input file was created using the BEAUti v2.7.5 software. In GMYC, an ultrametric tree was estimated using BEAST v2.7.7 software with a relaxed molecular clock model, the GTR Gamma substitution model, and a birth-death speciation process on an arbitrary timescale (Bouckaert et al. 2019; Heled 2015). The Markov Chain Monte Carlo (MCMC) method was employed, a random UPGMA tree was used as the starting tree, and the dataset was run for 10 million generations with sampling every 1,000 generations. Effective sample sizes exceeding 200 were verified using the Tracer v1.7.2 software (Rambaut et al. 2018). Trees generated by BEAST v2.7.7 were summarized using TreeAnnotator v2.7.7 software, incorporating 10% burn-in, a posterior probability of 0.0 and median node heights to produce a maximum clade credibility tree. GMYC analyses were conducted using R v4.3.3 and R-Studio v12.0 software (Paradis 2012).

Bouckaert, R., Vaughan, T. G., Barido-Sottani, J., Duchêne, S., Fourment, M., Gavryushkina, A., Heled, J., Jones, G., Kühnert, D., & De Maio, N. (2019) BEAST 2.5: An advanced software platform for Bayesian evolutionary analysis. *PLoS Computational Biology* 15, e1006650.

Fujisawa, T. and Barraclough, T. G. (2013) Delimiting species using single-locus data and the Generalized Mixed Yule Coalescent approach: a revised method and evaluation on simulated data sets. *Systematic Biology* 62, 707-724.

Hall, T. A. (1999) BioEdit: a user-friendly biological sequence alignment editor and analysis program for Windows 95/98/NT. *Nucleic Acids Symp. Ser.*, *41*, 95-98.

Paradis, E. (2012) *Analysis of Phylogenetics and Evolution with R* (Vol. 2). Springer.

Puillandre, N., Brouillet, S. and Achaz, G. (2021) ASAP: assemble species by automatic partitioning. *Molecular Ecology Resources* 21, 609-620.

Rambaut, A., Drummond, A. J., Xie, D., Baele, G., & Suchard, M. A. (2018) Posterior summarization in Bayesian phylogenetics using Tracer 1.7. *Systematic Biology 67*(5), 901-904.

**GMYC Results**

method: single

likelihood of null model: 33.55941

maximum likelihood of GMYC model: 34.38242

likelihood ratio: 1.646019

result of LR test: 0.4391082n.s.

number of ML clusters: 2

confidence interval: 1-3

number of ML entities: 7

confidence interval: 1-8

threshold time: -0.00324391

**GMYC-predicted species**

**Species 1:** 249-Austerdalsbreen, 256-Engabreen

**Species 2:** Seq1Decept, Seq2Decept

**Species 3:** 115-Larsbreen

**Species 4:** 117-Larsbreen

**Species 5:** 42-Foxfonna

**Species 6:** 44-Foxfonna

**Species 7:** Seq3Decept


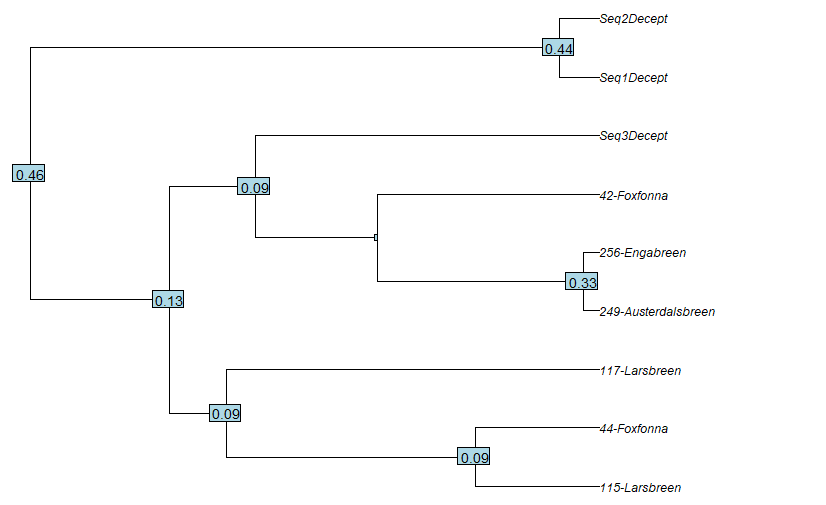


**Figure 3.** Species delimitation of Mt. Deception bdelloid rotifers and their congeners using GYMC. Specimens from the designated clade SP05 (Shain et al. 2024) comprise Mt. Deception and Norwegian bdelloids. Seven OTUs (operational taxonomic units) were predicted by GMYC analysis (Fujisawa and Barraclough 2013), including two OTUs from Mt. Deception (variants 1/2 and 3, respectively).

**ASAP Method**

| **Species number** | **Sequences** |
| --- | --- |
| **Species-1** | Seq1Decept |
|  | Seq2Decept |
| **Species-2** | Seq3Decept |
| **Species-3** | 42-Foxfonna |
| **Species-4** | 249-Austerdalsbreen |
|  | 256-Engabreen |
| **Species-5** | 44-Foxfonna |
|  | 115-Larsbreen |
| **Species-6** | 117-Larsbreen |


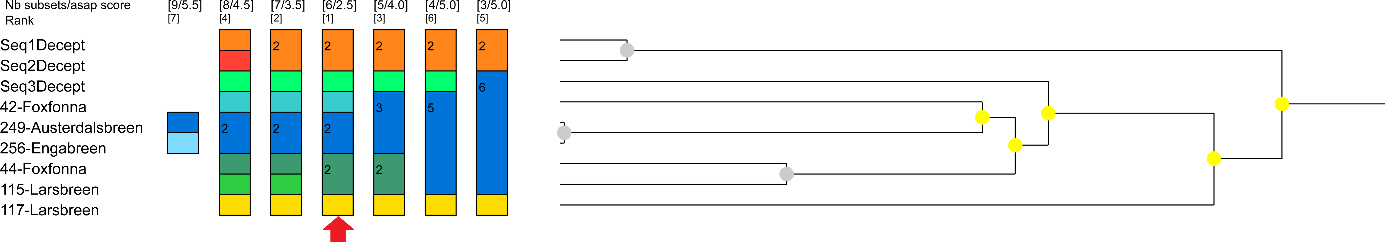


**Figure 4.** Species delimitation of Mt. Deception bdelloid rotifers and their congeners using ASAP. Specimens from the designated clade SP05 (Shain et al. 2024) comprise Mt. Deception and Norwegian bdelloids. Six OTUs (operational taxonomic units) were predicted by ASAP analysis (Puillandre et al. 2021), including two OTUs from Mt. Deception (variants 1/2 and 3, respectively).


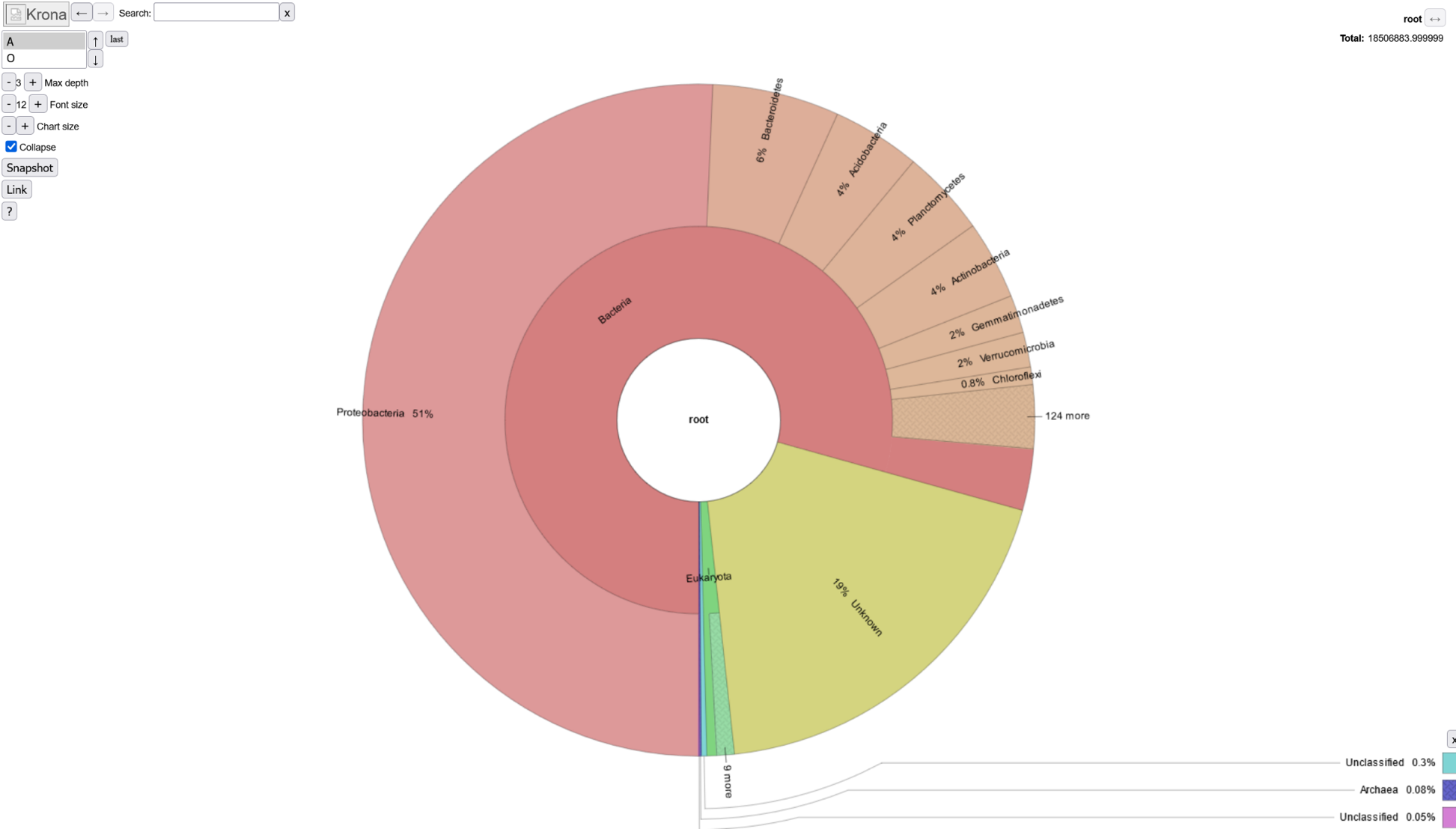


**Figure 5.** Metagenome analysis of Mt. Deception beaker aqueous and sediment combined fractions. All three domains of life were represented: Eubacteria (~78%), Eukarya (1.5%), Archaea (0.09%), unknown (19.5%).

**Table 1.** Distance matrix between individual rotifers at the COI locus (black text) with standard errors (blue text).

|  | **1** | **2** | **3** | **4** | **5** | **6** | **7** | **8** | **9** |
| --- | --- | --- | --- | --- | --- | --- | --- | --- | --- |
| **Mt. Dec1** | - | 0.003 | 0.014 | 0.015 | 0.014 | 0.013 | 0.017 | 0.014 | 0.014 |
| **Mt. Dec2** | 0.004 | - | 0.014 | 0.014 | 0.014 | 0.012 | 0.017 | 0.013 | 0.013 |
| **Mt. Dec3** | 0.072 | 0.066 | - | 0.012 | 0.012 | 0.010 | 0.012 | 0.009 | 0.009 |
| **42-Foxfonna** | 0.072 | 0.066 | 0.047 | - | 0.012 | 0.011 | 0.013 | 0.009 | 0.009 |
| **44-Foxfonna** | 0.066 | 0.061 | 0.050 | 0.050 | - | 0.006 | 0.014 | 0.009 | 0.009 |
| **115-Larsbreen** | 0.058 | 0.052 | 0.040 | 0.047 | 0.016 | - | 0.011 | 0.009 | 0.009 |
| **117-Larsbreen** | 0.092 | 0.086 | 0.055 | 0.066 | 0.066 | 0.048 | - | 0.012 | 0.012 |
| **249-Austerdalsbreen** | 0.063 | 0.058 | 0.035 | 0.030 | 0.032 | 0.032 | 0.055 | - | 0.000 |
| **256-Engabreen** | 0.063 | 0.058 | 0.035 | 0.030 | 0.032 | 0.032 | 0.055 | 0.000 | - |
